# The mental health of young people who are not in education, employment, or training: a systematic review and meta-analysis

**DOI:** 10.1007/s00127-021-02212-8

**Published:** 2021-12-21

**Authors:** Geneviève Gariépy, Sofia M. Danna, Lisa Hawke, Joanna Henderson, Srividya N. Iyer

**Affiliations:** 1 Montreal Mental Health University Institute, Montreal, QC Canada; 2 School of Public Health, Department of Social and Preventive Medicine, University of Montreal, Montreal, QC Canada; 3 Douglas Research Centre, Montreal, QC Canada; 4 Centre for Addiction and Mental Health, Toronto, ON Canada; 5 ACCESS Open Minds (Pan-Canadian Youth Mental Health Services Research Network), Montreal, QC Canada; 6 Department of Psychiatry, McGill University, Montreal, QC Canada

**Keywords:** Education or employment, NEET, Youth mental health, Substance use, Systematic review, Meta-analysis

## Abstract

**Purpose:**

There are increasing concerns about the intersection between NEET (not in education, employment, or training) status and youth mental ill-health and substance use. However, findings are inconsistent and differ across types of problems. This is the first systematic review and meta-analysis (PROSPERO-CRD42018087446) on the association between NEET status and youth mental health and substance use problems.

**Methods:**

We searched Medline, EMBASE, Web of Science, ERIC, PsycINFO, and ProQuest Dissertations and Theses (1999–2020). Two reviewers extracted data and appraised study quality using a modified Newcastle–Ottawa Scale. We ran robust variance estimation random-effects models for associations between NEET and aggregate groups of mental ill-health and substance use measures; conventional random-effects models for associations with individual mental/substance use problems; and subgroup analyses to explore heterogeneity.

**Results:**

We identified 24 studies from 6,120 references. NEET status was associated with aggregate groups of mental ill-health (OR 1.28, CI 1.06–1.54), substance use problems (OR 1.43, CI 1.08–1.89), and combined mental ill-health and substance use measures (OR 1.38, CI 1.15–1.64). Each disaggregated measure was associated with NEET status [mood (OR 1.43, CI 1.21–1.70), anxiety (OR 1.55, CI 1.07–2.24), behaviour problems (OR 1.49, CI 1.21–1.85), alcohol use (OR 1.28, CI 1.24–1.46), cannabis use (OR 1.62, CI 1.07–2.46), drug use (OR 1.99, CI 1.19–3.31), suicidality (OR 2.84, CI 2.04–3.95); and psychological distress (OR 1.10, CI 1.01–1.21)]. Longitudinal data indicated that aggregate measures of mental health problems and of mental health and substance use problems (combined) predicted being NEET later, while evidence for the inverse relationship was equivocal and sparse.

**Conclusion:**

Our review provides evidence for meaningful, significant associations between youth mental health and substance use problems and being NEET. We, therefore, advocate for mental ill-health prevention and early intervention and integrating vocational supports in youth mental healthcare.

**Supplementary Information:**

The online version contains supplementary material available at 10.1007/s00127-021-02212-8.

## Introduction

Transitioning from education into work is a milestone of emerging adulthood that about one in seven young people in economically developed countries struggle to attain [1], falling into the category of NEET—not in education, employment, or training. Concerns over these youth are growing worldwide [2, 3]. The term NEET was coined in a 1999 report called “Bridging the Gap” from the United Kingdom [4]. By 2019, between 5.6% (Luxembourg) and 28.8% (Turkey) of 15 to 29-year-olds in Organisation for Economic Co-operation and Development countries were NEET [1]. Economic fallouts of the COVID-19 pandemic are expected to swell these numbers [3], as even early on in the pandemic, data showed that NEET rates were higher in the second quarter of 2020 than the previous year in 45 out of 50 countries [5]. Youth who are NEET are considered vulnerable as they face social exclusion and disempowerment, and disproportionately come from disadvantaged backgrounds [6, 7]. Being outside school and the workforce limits their ability to gain skills and experience that could improve their prospects [8–10].

Being NEET is intertwined with mental health and substance use problems in young people. Studies have linked being NEET with the emergence of symptoms of depression, anxiety, substance use, and suicidality [11–15]. Conversely, mental health and substance use problems can deplete the drive and energy needed to enter the workforce or continue education/training and increase the risk of becoming NEET. However, the link between being NEET and poor mental health is unclear. Cross-sectional relationships are not always supported by longitudinal data [16, 17] and there are indications that the relationship differs by type of mental health or substance use problem [14, 18]. Furthermore, the association between being NEET and mental health problems may also differ in strength and significance across gender, depending on mental health problem [19, 20]. For instance, Henderson [19] found that the association between internalizing disorders and being NEET was significant in only men. For externalizing disorders, however, the association with NEET status was significant for both men and women.

Previous reviews have reported on the association between mental health problems and youth unemployment [21, 22] and school disengagement [23, 24], but none investigated youth disengaged from both work and school. One narrative review examined the correlates of being NEET [6], but with little in-depth information on mental health. A synthesis of the literature is needed to inform the discussion on the growing youth population who are NEET and its intersection with youth mental health and substance use problems. This information is also needed to develop intervention studies and effective strategies to promote youth engagement in employment, education, and training.

Our primary objective was therefore to systematically review and synthesize via meta-analysis the literature on the associations between being NEET and mental health and substance use problems among youth. We expected NEET status to relate to mental ill-health measures, substance use measures, and all measures of mental ill-health and substance use combined; and the associations to vary across mental health and substance use problems. Our review thus extends the literature by focussing on youth disengagement from both education and employment and by examining the strength and consistency of associations across types of mental health and substance-use problems. Our secondary objectives were to investigate the directionality of the associations from longitudinal data and to examine subgroup differences by gender, age, and population-based versus clinical samples. We expected the association between NEET status and mental health and substance use problems to be bidirectional and to differ in strength by gender. We were agnostic as to differences by age and sample type.

## Methods

### Search strategy

We followed MOOSE reporting guidelines [25]. We searched Medline, EMBASE, ISI Web of Science, ERIC, PsycINFO, and ProQuest Dissertations and Theses Online from January 1, 1999 to May 2020, imposing no language restriction (see MEDLINE search strategy in Supplementary Appendix 1). We limited searches to the last 20 years because our objective was to synthesize contemporary knowledge of policy and practice relevance. The study is registered through PROSPERO (CRD42018087446) [26].

### Selection criteria

We included observational studies with individual-level data that estimated the association between being NEET and mental and/or substance use symptoms or disorders among persons aged 15–34 years. We chose this age range to accommodate internationally diverse definitions of youth [27]. Studies had to identify NEET status by explicitly querying work and education or training status. Measures included those for any specific or general mental or substance use disorder; psychological or behavioural problems; psychological distress or well-being; or suicidality, measured on a dichotomous or continuous scale of symptoms, severity, or score. We excluded neurodevelopmental disorders and disabilities typically diagnosed in childhood (e.g., autism, intellectual disability) since we expected developmental and learning problems to have a unique association with becoming NEET. We excluded abstracts but considered unpublished studies if information was available for data extraction and quality assessment. We searched references of primary studies and review articles for additional studies. All references were uploaded to Covidence software [28].

The screening of titles and abstracts followed by screening of full texts for inclusion and exclusion criteria; data extraction from eligible studies (see data extraction form in Supplementary Appendix 2); and quality assessment of studies using a modified Newcastle–Ottawa Scale [29] (see description in Supplementary Appendix 3) were done independently by two reviewers, including first author/epidemiologist GG and either a psychiatry graduate student or a research assistant with a Master’s in mental health epidemiology (SD). Disagreements were resolved by consensus or by author SI. We emailed study authors for further information where necessary.

### Data analysis

We conducted meta-analysis to quantitatively synthesize the literature. We ran robust variance estimation (RVE) random-effects models to obtain associations between NEET status and three aggregate groups; namely, mental ill-health (comprised of psychological distress, mood disorders, anxiety disorders, and behavioural disorders); substance use problems (comprised of alcohol, cannabis disorder, and drug use disorders); and all measures of mental ill-health and substance use problems taken together (comprised of the measures included in the mental ill-health and substance use groups, any other disorder, and suicidality). RVE allowed us to pool statistically dependent estimates (i.e., multiple estimates that are correlated because they arise from the same participant samples) into estimates incorporating all relevant measures for these aggregated groups without having to know or specify their covariance structures. Additionally, our analyses benefit from small-sample correction, which has been argued as necessary to implement when using RVE hypothesis testing [30]. It is important to note that, among small samples, hypothesis testing using RVE requires degrees of freedom be greater or equal to four to be accurate. Below four degrees of freedom, the *t* distribution approximation on which testing is based no longer holds, and the type I error will be greater than indicated by the *p* value being used [31]. We also conducted conventional random-effects models to obtain associations between NEET status and individual mental health and substance use problems. Forest plots were generated to display all main meta-analyses described above.

Our pooled results should be interpreted cautiously, given the highly heterogeneous study methodologies. We used the odds ratio (OR) as a summary measure since most studies with available quantitative data reported ORs. When multiple studies used the same dataset, we only included the study with the largest sample size to avoid double counting. For studies that only provided gender-stratified results, we combined the results using a fixed-effects model to include in the main meta-analysis. For studies that only reported a *p* value < 0.001, we calculated a confidence interval (CI) assuming a conservative *p* value of 0.001. For studies reporting only a *p* value > 0.05, we assumed a conservative *p* value of 0.10. All intervals reported are 95% CIs.

To explore sources of heterogeneity, we conducted subgroup analyses by gender, age group (< 18 vs ≥ 18 years old), and sample type (population-based vs clinical). Moreover, we investigated the potential directionality of associations from longitudinal studies that examined NEET status as a predictor of later mental health and substance use problems, and studies that examined the inverse relationship. We used fixed-effects models for subgroup analyses because there were too few studies by subgroup to estimate between-study variance with precision [32]. Study heterogeneity was evaluated using the *I*^2^ index. We did not assess publication bias using quantitative methods because these are not recommended under conditions of high heterogeneity [33]. Meta-analyses were conducted in R (v3.6.1) using the meta, metafor, and robumeta packages [30, 34, 35].

## Results

**Fig. 1:**
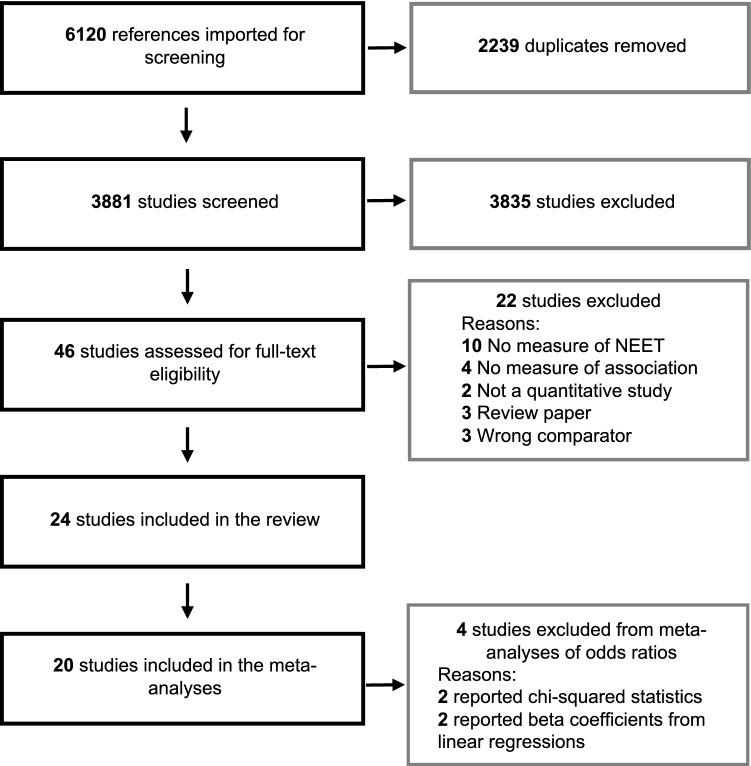
PRISMA flow diagram

From 6120 identified references, we included 24 studies (see PRISMA flow diagram in Fig. 1), which represented 548,862 unique individuals from the UK (*k* = 6 studies); Australia (*k* = 4, two using the same sample); Mexico (*k* = 3, all using the same sample); Sweden (*k* = 3); Italy (*k* = 2) [13, 36]; Canada (*k* = 2); Brazil (*k* = 1); Norway (*k* = 1); Ireland (*k* = 1); Switzerland (*k* = 1); and Greece (*k* = 1). Study characteristics, detailed study findings, and quality assessment ratings appear in Table 1 and Supplementary Appendices 4 and 5, respectively. For 11 studies, the age range of the sample at baseline was under 18 years; three studies only included samples above the age of 19; and 10 studies included samples that were both below and above 18 years of age (e.g., 15–25). Cohort studies were most common (*k* = 13), followed by cross-sectional (*k* = 10) and case–control (*k* = 1) studies. Being NEET was associated with at least one measure of mental health or substance use problems in 75% of studies (18/24). Study quality was low in five studies; moderate in nine studies; and high in 10 studies. Measures of mental health and substance use problems included problems or symptoms of mood (*k* = 12), anxiety (*k* = 11), behaviour (*k* = 8), alcohol use (*k* = 9), cannabis use (*k* = 6), and drug use (*k* = 8) disorders, general psychological distress (*k* = 9), suicidal behaviours (*k* = 7), and any psychiatric disorder (*k* = 5).

**Table 1:** Characteristics of the selected studies

References	Country, baseline year	Design	*N*	Sample	NEET measure	Mental health and substance use measures	Direction(s) of association^a^	Statistical Methods	Covariates^b^
Baggio et al. [17]	Switzerland, 2010–2012	Cohort	4758	Young men in their early 20's at baseline	Current status, excluding those in the military or civic service	General mental health, depressive symptoms, alcohol use, and cannabis use disorder	MH < – > NEET NEET– > MH MH– > NEET	Logistic regression	Language, age, family SES
Bania et al. [42]	Norway, 2003–2005	Cohort	3987	Youth 15–16 years old at baseline	Did not complete post-secondary school and unemployed for 1 + year, or received 6 + months of sickness benefits during the 9-year study period (2003–2012)	Conduct and emotional problems	MH– > NEET	Logistic regression	Gender, residency, ethnicity, parental education
Basta et al. [46]	Greece, 2016	Cross-sectional	2771	Youth 15–24 years old	Current status	Anxiety and depressive symptoms, drug use	MH < – > NEET	Logistic regression	Gender, insurance, income, living with parents, being married, financial support by others
Benjet et al. [12]	Mexico, 2005	Cross-sectional	3005	Youth 12–17 years old	Current status	Mood, anxiety, substance use and behavioural disorders, and suicidal behaviours	MH < – > NEET	Logistic regression	Age, gender, SES, marital status, having children, living with parents, parental SES
Bynner and Parsons [20]	United Kingdom, 1987	Cohort	930	Youth 16–18 years old at baseline not in full-time education	NEET for 6 + months between 16–18 years old	Psychological distress	NEET– > MH	Logistic regression	Birth weight, cognitive abilities, hobbies, and family circumstances in childhood
Cairns et al. [37]	Australia, 2013	Cross-sectional	226	Youth 15–25 years old seeking services	Current status, excludes those in carer role	Psychological distress, history of mental health diagnosis, and history of illicit drug use	MH < – > NEET	Multinomial logistic regression	Age, gender, secondary school dropout, psychological distress, history of mental health diagnosis, history of illicit drug use
Gariépy and Iyer [40]	Canada, 2014	Cross-sectional	5622	Youth 15–29 years old	Current status	Depression, bipolar, generalized anxiety, alcohol use, cannabis use, and drug use disorders, and suicidal behaviours	MH < – > NEET	Logistic regression	Age, gender, SES, ethnicity, immigrant status, living arrangement, health conditions, location
Goldman-Mellor et al. [14]	United Kingdom, 2012–2013	Cohort	2232	Twins 17–18 years old at baseline	Current status, excludes parents	Depressive, generalized anxiety, alcohol, cannabis, and conduct disorders (age 18), and childhood depressive, anxiety, substance use disorders, and suicidal behaviours (age 12)	MH– > NEET MH < – > NEET	Modified Poisson and logistic regression	Gender, cognitive ability, family SES, neighborhood, childhood mental health problems
Gutierrez-Garcia et al. [15]	Mexico, 2005	Cohort	1071	Youth 12–17 years old at baseline	Current status	Mood, anxiety, alcohol use, substance use and behavioural disorders, and suicidal behaviours	NEET– > MH	Logistic regression	Gender, age, marital status, family SES
Gutierrez-Garcia et al. [39]	Mexico, 2013	Cross-sectional	1071	Youth 19–26 years old	Current status	Mood, anxiety, behavioural, and substance use disorders; suicidal behaviours	MH < – > NEET	Logistic regression	Gender, marital status, has children, education, living with family of origin
Hale and Viner [49]	United Kingdom, 2004	Cohort	8682	Youth 13 years old at baseline	Current status	Psychological distress	MH– > NEET	Logistic regression	SES, ethnicity and educational attainment
Hammerton et al. [43]	Brazil, 2004–2005 United Kingdom, 2002–2003	Cohort	Brazil: 3939; UK: 5079	Youth age 11 years old at baseline	Current status	Conduct problem and oppositional problem	MH– > NEET	Logistic regression	Sex, parental separation, fear of the neighborhood at age 11, maternal risk factors (SES, smoking, depression, unplanned pregnancy, alcohol use, urinary infection during pregnancy), birth factors (intrauterine growth restriction, gestational age, premature birth)
Henderson et al. [19]	Canada, 2009–2013	Cross-sectional	2576	Youth 12–24 years old seeking services	Current status	Internalizing, externalizing, and substance use disorders	MH < – > NEET	Chi-square tests	Age
López-López et al. [41]	United Kingdom, 2002–2003	Cohort	4501	Youth age 11 years old at baseline	Current status	Trajectories of depressive symptoms	MH– > NEET	Logistic regression	Sex, IQ, maternal postnatal depression, maternal education, attitude towards school and academic results at age 11
Manhica et al. [48]	Sweden, 2005–2009	Cohort	485,839	Youth 19–24 years old with secondary education	NEET indicator of labour market attachment in past year	Alcohol use disorder	NEET– > MH	Cox regression	Sex, age, domicile and origin
Nardi et al. [13]	Italy, 2010–2011	Case–control	228	NEET youth from juvenile court services or the community and non-NEET youth from a technical institute, 16–23 years old	Not stated	Symptoms of nervousness, mood swings, or thoughts of suicide	MH < – > NEET	Chi-square tests	None
Nardi et al. [36]	Italy, 2010–2011	Cross-sectional	143	Youth involved in criminal proceedings, 16–19 years old	Current status	Psychiatric disorders diagnosed by psychiatric services	MH < – > NEET	Chi-square tests	None
O'Dea et al. [38]	Australia, 2011–2012	Cross-sectional	676	Youth seeking services 15–25 years old	Current status	Symptoms of mood, anxiety, alcohol use, and cannabis use disorders	MH < – > NEET	Logistic regression	Age, gender, criminal charges, economic hardship, self-rated disability, clinical stage
O'Dea et al. [16]	Australia, 2011–2012	Cohort	448	Youth seeking services 15–25 years old at baseline	Past-month status	Depressive and generalized anxiety disorders	MH– > NEET NEET– > MH	Multinomial logistic regression and chi-square tests	Age, gender, location, immigrant background
Power et al. [18]	Ireland, 2000–2002	Cohort	168	Youth 15–25 years old	Not stated	Mood, anxiety, alcohol, substance use disorders, and suicidal behaviours	MH < – > NEET MH– > NEET	Logistic regression	Gender and SES
Rodwell et al. [57]	Australia, 1992–1993	Cohort	1938	Youth 14–15 years old at baseline	Current status	Common mental disorders; disruptive, alcohol use, and cannabis use disorders	MH– > NEET	Logistic regression	Gender, marital status, location, parental education, year
Stea et al. [50]	Sweden	Cross-sectional	480	NEET youth from vocational services and non-NEET youth attending high school, 16–21 years old	Current status	Psychological distress	MH < – > NEET	Logistic regression	Sex, parental education
Stea et al. [45]	Sweden	Cross-sectional	480	NEET youth from vocational services and non-NEET youth attending high school, 16–21 years old	Current status	Cannabis use	MH < – > NEET	Logistic regression	Sex, age, parental education
Symonds et al. [11]	United Kingdom, 2005	Cohort	11,082	Youth 14–15 years old at baseline	Current status, excludes those caring full-time for others	Symptoms of depression, anxiety, and positive mental functioning	MH– > NEET	Linear regression	Gender, ethnicity, SES, childhood achievement

^a^NEET: not in education, employment, or training; MH: mental health

^b^SES: socioeconomic status

4 out of the 24 studies were excluded from meta-analyses of odds ratios because two reported chi-squared statistics [13, 36] and 2 reported beta coefficients from linear regressions [11, 37]. Further, two pairs of studies used the same data to calculate the same estimates [15, 16, 38, 39]. We only included one study from each pair in the RVE meta-analyses [38, 39].

The meta-analyses found significant associations between NEET and all three aggregated groups, i.e., mental health problems (*k* = 15 studies; *n* = 25 effect sizes, OR 1.28, CI 1.06–1.54; see Supplementary Appendix 6 for forest plot); substance use problems (*k* = 11, *n* = 16, OR 1.43; CI 1.08–1.89; see Supplementary Appendix 7 for forest plot); and all measures of mental health problems, substance use problems, and suicidality combined (*k* = 18, *n* = 48, OR 1.38; CI 1.15–1.64).

**Fig. 2:**
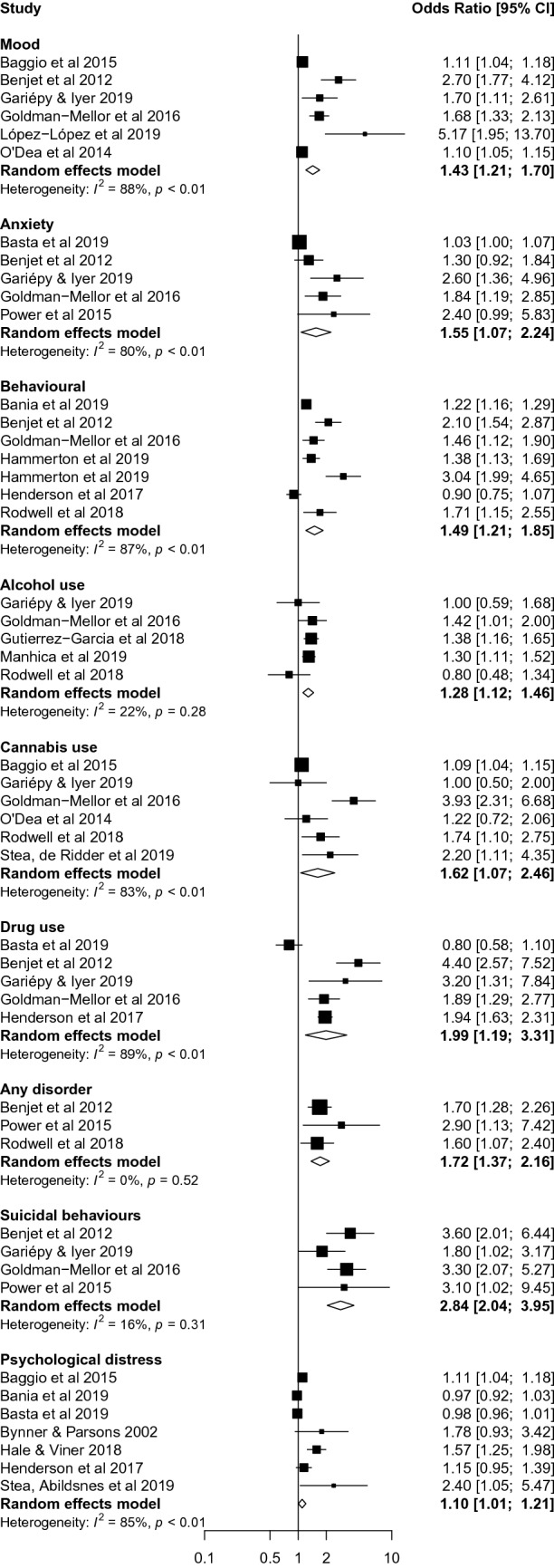
Forest plot from the meta-analysis disaggregated by mental-ill health, substance use problems and suicidality measures

Table 2 presents summaries of findings by type of mental health or substance use problem and Fig. 2 presents forest plot by each type of problem. The evidence most consistently pointed to an association between NEET and symptoms of mood disorders [12, 14, 17, 38, 40, 41] (*k* = 6 non-overlapping studies; OR 1.43, CI 1.21–1.70); behavioural disorders [12, 14, 19, 42–44] (*k* = 6; OR 1.49, CI 1.21–1.85); cannabis use problems [14, 17, 38, 40, 44, 45] (*k* = 6; OR 1.62, CI 1.07–2.46); drug use problems [12, 14, 19, 40, 46] (*k* = 5; OR 1.99, CI 1.19–3.31); any psychiatric disorder [12, 18, 44] (*k* = 3; OR 1.72, CI 1.37–2.16); and suicidal behaviours [12, 14, 18, 40] (k = 4; OR 2.84, CI 2.04–3.95).

**Table 2:** Summary of findings by type of mental health and substance use disorder

Type	All studies		Studies reporting odds ratios				Studies reporting beta coefficients		Studies reporting Chi-square statistics	
	Number of studies	% studies reporting a statistically significant association	Number of non-overlapping studies	Number of excluded studies^a^	Pooled OR (95% CI)	*I*^2^ (%)	Number of studies	Beta coefficient (95% CI)	Number of studies	*p* value
Mood	12	75% (9/12)	6	3	1.43 (1.21, 1.70)	88.0	1	*β* 0.10 (*p* < 0.05)^†^	1	*p* > 0.05^‡^
Anxiety	10	40% (4/10)	5	3	1.55 (1.07, 2.24)	79.5	1	*β* 0.10 (*p* < 0.05)^†^	1	*p* > 0.05^‡^
Behavioral	8	63% (5/8)	6	2	1.49 (1.21, 1.85)	87.0	0	–	0	–
Alcohol use	9	33% (3/9)	6	3	1.28 (1.12, 1.46)	21.7	0	–	0	–
Cannabis use	6	66% (4/6)	6	0	1.62 (1.07, 2.46)	83.4	0	–	0	–
Drug use	8	75% (6/8)	5	3	1.99 (1.19, 3.31)	89.4	0	–	0	
Any disorder	5	90% (4/5)	3	1	1.72 (1.37, 2.16)	0.0	0	–	1	*p* > 0.05^‡^
Suicidal behaviors	7	71% (5/7)	4	2	2.84 (2.04, 3.95)	15.6	0	–	1	*p* > 0.05^‡^
Psychological distress	9	33% (4/12)	7	1	1.04 (0.96, 1.14)	82.7	1	*β* 0.06 (*p* < 0.05)^†^	0	–

^a^Studies with overlapping data or with specific OR not reported

^†^95% CI or specific *p* value not reported

^‡^Specific *p* value not reported

The evidence was more mixed—with fewer than 50% of non-overlapping studies reporting a significant finding—for NEET status being associated with anxiety disorders [12, 14, 18, 40, 46] (*k* = 5 non-overlapping studies; OR 1.55, CI 1.07–2.24); alcohol use problems (*k* = 5; OR 1.28, CI 1.12–1.46); and psychological distress (*k* = 7; OR 1.10, CI 1.01–1.21). Results were similar when we excluded low quality studies.

### Sub-group analyses

For each of the three aggregate groups, subgroup analyses were conducted for directionality, age, and gender. There was evidence of mental health problems (first aggregated group; *k* = 8 studies; *n* = 12 effect sizes, OR 1.33, CI 1.01–1.74) and all measures combined (third aggregated group; *k* = 9; *n* = 19, OR 1.39, CI 1.03–1.86) being associated with subsequent NEET status. Other subgroup analyses conducted within the three aggregated groups had too few degrees of freedom (*df* < 4) to be reliable, so results should be considered exploratory (Supplementary Appendix 8).

#### Directionality of association

Longitudinal data provided some evidence for bidirectional associations (see Supplementary Appendix 9 for summary of significant findings of studies by directionality of association and type of mental or substance use disorder or symptoms). Ten studies measured symptoms of mental ill-health and/or substance use problems before the emergence of NEET status. Symptoms of mood disorders [14, 16, 17, 41] (*k* = 4 non-overlapping studies; OR 1.12, CI 1.07–1.18); behavioural problems [42–44] (*k* = 3; OR 1.25, CI 1.19–1.32); cannabis use problems [17, 44] (*k* = 2; OR 1.10, CI 1.04–1.15); drug use problems [14] (*k* = 1; OR 1.89, CI 1.29–2.77); any mental disorder [18, 44] (*k* = 2; OR 1.83, CI 1.27–2.62); and suicidal behaviours [14] (*k* = 1; OR 3.30, CI 2.07–5.27) were associated with later NEET status. Alcohol use disorder [17, 44] was not so associated (*k* = 2; OR 0.80, CI 0.48–1.34), and evidence was equivocal for anxiety symptoms/disorders [14, 16] (*k* = 2; OR 1.38, CI 0.81–2.36) and psychological distress [11, 17, 42, 47] (*k* = 4; OR 1.04, CI 1.00–1.08).

Five studies examined NEET status prior to mental health and substance use outcomes. NEET status predicted later suicidal behaviours in a single study [15] (*k* = 1; OR 2.40, CI 1.32–4.31); symptoms of mood disorder in one study [15] (*k* = 1; OR 1.67, CI 1.12–1.90), but not another [16] (*k* = 1; OR 1.94, CI 0.17–21.60); and alcohol use disorder in two studies [15, 48] (*k* = 2; OR 1.22, CI 1.12–1.32), but not another [17] (*k* = 1; *p* > 0.05, values unavailable). Being NEET did not predict later symptoms of anxiety [15, 16] (*k* = 1; OR 0.40, CI 0.10–1.65); behavioural problems [15] (*k* = 1; OR 0.83, CI 0.45–1.50); cannabis use [17] (*k* = 1; *p* > 0.05, values unavailable); drug use problems [15] (*k* = 1; OR 1.03, CI 0.71–1.50); or psychological distress [9, 17] (*k* = 2; OR 1.78, CI 0.93–3.42).

#### Associations by gender

Six studies conducted gender-stratified analysis [19, 20, 42, 46, 49, 50]. In Bania et al. [42], conduct problems at age 15–16 predicted becoming NEET 9 years later among men (OR 1.17, CI 1.07–1.28) and women (OR 1.25, CI 1.17–1.33), while emotional problems were associated with lower odds of becoming NEET in men (OR 0.88, CI 0.81–0.97), but not women (OR 1.04, CI 0.97–1.11).

Hale and Viner [49] found that psychological distress at age 13 predicted being NEET at age 19 among men (OR 1.72, CI 1.24–2.41) and women (OR 1.49, CI 1.11–1.99). Bynner and Parsons [20] found that being NEET at age 16–18 did not predict psychological distress at age 21 in men (OR 2.20, *p* > 0.05, CI unavailable) and women (OR 1.69, *p* > 0.05).

In their cross-sectional study, Stea et al. [50] found an association between NEET and psychological distress among women (OR 2.40, CI 1.00–5.20), but not men (estimate unavailable), whereas Basta et al. [46] found no association with distress among women (OR 0.98, CI 0.95–1.02) and men (OR 0.99, CI 0.96–1.03).

Basta et al. [46] found an association between anxiety problems and NEET among women (OR 1.05, CI 1.01–1.10), but not men (OR 1.01, CI 0.96–1.03). In a cross-sectional study, Henderson et al. [19] found that being NEET was associated with substance misuse among men (OR 1.83, CI 1.43–2.34) and women (OR 2.05, CI 1.58–2.66); externalizing disorders among both men (OR 0.93, CI 0.73–1.19) and women (OR 0.87, CI 0.67–1.12); and internalizing symptoms among men (OR 1.39, CI 1.08–1.78), but not women (OR 1.08, CI 0.80–1.45).

#### Associations by age

We compared findings for participants who were < 18 years (*k* = 4 studies) [12, 19, 20, 42] and ≥ 18 years old (*k* = 7) [14, 17–19, 39, 44, 49]. The association was consistent among younger (< 18 years) youth between NEET status and mood problems [11, 12] (beta coefficient 0.0710, *p* < 0.05 in one study; OR 2.70, CI 1.77–4.12 in the other study); behavioural problems [12, 19, 42] (*k* = 3; OR 1.24, CI 1.18–1.30); and drug use problems (*k* = 2; OR 1.69, CI 1.38–2.07). Results were weaker for psychological distress [19, 20, 42] (*k* = 3; OR 0.97, CI 0.92–1.03) and anxiety problems [11, 12] (*k* = 2; OR 1.30, CI 0.92–1.84 in one study; beta coefficient = 0.07, *p* > 0.05 in other study).

In youth ≥ 18 years old, there was an association with anxiety disorders [14, 18, 39] (*k* = 3; OR 1.59, CI 1.12–2.26), behavioural disorders [14, 19, 39, 44] (*k* = 4; OR 1.32, CI 1.12–1.55); cannabis use problems (*k* = 3; OR 1.11, CI 1.05–1.16); any disorder [18, 44] (*k* = 2; OR 1.76, CI 1.21–2.54); and general psychological distress (*k* = 3; OR 1.15, CI 1.08–1.22).

In youth ≥ 18 years old, evidence was mixed for symptoms of mood disorder [14, 17, 18, 39] (*k* = 4; OR (*n* = 3) 1.14, CI 1.07–1.21; and missing OR with *p* > 0.05, CI unavailable in other study); drug use disorders [14, 19, 39, 44] (*k* = 4; OR (*n* = 3) 1.99, CI 1.61–2.45; and missing OR with *p* > 0.05, CI unavailable in other study); and alcohol use problems [14, 17, 18, 39, 44] (*k* = 5; OR (*n* = 3) 1.32, CI 1.14–1.54; missing OR with *p* > 0.05, CI unavailable in other studies).

#### Associations by sample type

Similar patterns of association emerged between studies using clinical [16, 19, 37, 38] (*k* = 4 studies) and population-based samples [11, 12, 15, 17, 18, 20, 39–44, 46, 48, 49] (*k* = 18).

In clinical studies, service-seeking youth were more likely to be NEET if they presented with mood disorders [16, 38]; current [19] or past [37] drug use disorders; or co-occurring mental health problems [19]; but not if they had problems with alcohol or cannabis use [16, 38], anxiety disorders [16, 38], psychological distress [37], externalizing problems [19], or a history of any mental health diagnosis [37].

## Discussion

This is the first comprehensive systematic review and meta-analysis on the association between NEET status and mental health and substance use problems in youth. Being NEET was associated with mental health problems, substance use problems, and all measures combined in aggregate analyses. When disaggregated, NEET was most consistently associated with suicidal behaviours, drug use problems, any psychiatric disorders, cannabis use problems, behavioural problems, and mood problems. Findings for the association between NEET and anxiety problems, alcohol use, and psychological distress were mixed. Results were generally consistent across clinical and population-based samples but mixed across gender. These associations were particularly consistent among younger youth (< 18 years old). Longitudinal data indicated that mental health problems in early youth predicted a later NEET status, while evidence for the inverse relationship was equivocal and sparse. Together, these results point to early youth as a sensitive period for mental health and substance use problems becoming related with being NEET and increasing the vulnerability to later becoming NEET.

The aggregate analyses showed meaningful and significant associations between mental health and substance use problems in youth and being NEET. Although the overall evidence is based on a relatively limited and heterogeneous body of literature, the studies were generally of moderate to high quality. These results align with previous reviews on youth unemployment [21, 22] and school dropout [23, 24] that report a close connection between vocational disengagement and poor mental health. Our review extends this literature by focussing on youth disengagement from both education and employment and by revealing that the strength and consistency of associations vary across types of mental health and substance-use problems.

In our study, meta-analytical evidence from longitudinal data suggested that mental health problems and all measures of mental health and substance use (combined) predicted becoming NEET later. This evidence for their increased risk of becoming NEET aligns with the well-documented [24] drain of mental and substance use disorders on youths’ ability to perform at school and work. Disengagement from school and work may further disadvantage those with mental health problems, widening the gap between them and peers who follow more engaged developmental trajectories. Disengagement may also further heighten feelings of shame, hopelessness, and social exclusion [20, 51]. Analyses for NEET status predicting the later occurrence of mental health problems aggregated, substance use problems aggregated, and all measures combined were not conclusive. Nonetheless, there was evidence for NEET status predicting individual mental health/substance use problems, suggesting that being out of school and work, especially in early youth, could lead to mental health and substance use problems. Regardless of the directionality, school and work can provide crucial structures and experiences that enhance feelings of belonging, productivity, and hope for the future [52].

Contrary to our hypothesis, we discerned no clear gender-based pattern in the link between mental health problems and being NEET, although the evidence base was limited and most gender-stratified studies focussed on psychological distress. Nonetheless, there is evidence that the experience of being NEET could vary by gender. For instance, young women who are NEET are more likely to be stay-at-home parents or caretakers [53, 54]. Further, the consequences of being NEET may differ by gender. A British longitudinal study [20] found that, for young men, being NEET mainly impacted their job prospects, while for young women, it further affected their psychological well-being. To develop tailored strategies to prevent youth from becoming NEET or developing mental health problems when NEET, further research is needed into the intersections between gender and other subgroupings of vulnerability, NEET status, and mental illness.

Our review was constrained by the heterogeneity of mental health measures used in the reviewed studies, ranging from specific disorder subtypes (e.g., generalized anxiety disorder) to broad categories (e.g., any anxiety disorder) and general symptom scales. Many mental disorders like psychosis, eating disorders, and personality disorders, were not represented and few studies reported on comorbidity [19]. Divergent definitions of NEET status also limited comparability. Non-paid work like parenting counted as employment in some studies [14, 44], but not others. Most studies measured current NEET status, but some used timeframes from 1 month [16] to 9 years [42]. By assessing NEET status but not its duration, almost all studies captured the association of both short- and long-term vocational disengagement with mental health and substance use problems. To formulate more effective interventions and policies, research into the duration of NEET status and its association with mental and substance disorders is therefore needed. These definitional, methodological, and cultural challenges of measuring NEET status and the heterogeneity of its circumstances have also been previously noted [27, 54, 55].

We could not examine contextual/cultural influences on being NEET and mental health problems because the reviewed studies were from a limited number of specific geographical and political backgrounds. All the studies from this review were from Europe, North America, or Australia, with the exception of one study that included data from South America, limiting the generalizability of the evidence to other contexts like low- and middle-income countries. Furthermore, global and country-specific economic shifts may exacerbate associations between NEET status and mental-ill health and deserve exploration in the future. Evidence is from observational data thereby limiting direct causal inference. While we examined longitudinal studies to assess the potential directionality of association, none of the studies used specific panel regressions models and may therefore be biased by unobserved heterogeneity. Like other reviews, our findings may be affected by publication bias. While we could review papers in English, French, and Spanish, only English studies met our search criteria. We excluded studies that focussed on neurodevelopmental disorders or disabilities that are typically diagnosed in childhood. We recognize that these disorders could co-occur with mental and substance use disorders and contribute to being NEET and may even differ in their relationship with NEET compared to other mental disorders, and therefore should be examined in future work.

Notwithstanding these limitations, the studies provided data from diverse contexts and on a range of mental health and substance use outcomes, with generally consistent results despite methodological differences. Our review carefully assessed the association between being NEET and mental health and substance use problems, an emerging topic with important clinical and public health implications. We used rigorous methodology to search, systematically assess, and analyse current literature to explain our findings. Using RVE, we appropriately pooled multiple mental health measure estimates that were correlated because they came from the same participant samples. This allowed us to capture associations between NEET and overarching groups of mental health and substance use problems that reflect a generalized relationship between youth engagement and mental-ill health. In addition, we used subgroup analysis to investigate heterogeneity, directionality of association, and vulnerable subgroups.

We identified significant knowledge gaps in NEET and mental health research. First, the association between being NEET and mental health and substance use problems is likely context-sensitive and broadening the geographic ambit of studies is strongly recommended. Second, the association is likely marked by gender differences that bear teasing out. Third, rigorous research on the temporal relationship of mental disorders and being NEET is needed because the question of directionality remains unresolved. Moreover, the associations between duration and recurrence of NEET status and mental ill-health have yet to be systematically explored. Fourth, information about some mental disorders (e.g., psychosis) and comorbid disorders and their associations with being NEET is lacking. Finally, future research should include intervention studies to identify whether and for whom vocational and mental health supports are useful in averting and ending NEET status.

Realization of the loss to productivity and the wealth of nations from unaddressed youth mental health problems is increasing [56]. Although more longitudinal research is needed, our review found clear evidence for NEET status being a consequence of mental health problems and substance misuse. Efforts to prevent young people from becoming or remaining vocationally and socially disengaged should therefore include provisions for the prevention of and early intervention for mental health problems. Furthermore, because there is also evidence for a bidirectional relationship between NEET status and mental ill-health and because problems with vocational functioning are well-documented among youth with mental health problems [17, 57], youth mental health services should integrate educational and employment supports and services to address vocational needs and promote recovery.

The connectedness of vocational disengagement and mental health problems among young people underlines the need for consistent, widespread policy support for broader-spectrum integrated youth-focussed services [58, 59]. Our review also highlights the importance of schools, universities, and employers developing the will and capacity to address the needs of youth experiencing mental health problems. The socioeconomic disruptions and mental health implications of the ongoing pandemic make these needs ever more urgent. Our comprehensive synthesis can serve as a useful pre-pandemic reference point for future research on the associations between youth employment/education and mental health and substance use over the course of or after the COVID-19 pandemic.

## Supplementary Information

Below is the link to the electronic supplementary material.

Supplementary file1 (PDF 775 KB)

## References

[1] Youth not in employment, education or training (NEET) (indicator) (2021) https://data.oecd.org/chart/5MP5 Accessed 21 Apr 2021

[2] International Labour Office (2020). Global employment trends for youth 2020: technology and the future of jobs.

[3] Kassid S (2020) What about us? Youth (un)employment in times of COVID-19. World Future Council. https://www.worldfuturecouncil.org/covid19-what-about-us/. Accessed May 2020

[4] Social Exclusion Unit (1999). Bridging the gap: new opportunities for 16–18 year olds not in education, employment or training.

[5] Karkee V, Sodergren M-C (2021) How women are being left behind in the quest for decent work for all. International Labour Office Department of Statistics. https://www.ilostat.ilo.org/how-women-are-being-left-behind-in-the-quest-for-decent-work-for-all/. Accessed 29 Mar 2021

[6] Sadler K, Akister J, Burch S (2015). Who are the young people who are not in education, employment or training? An application of the risk factors to a rural area in the UK. Int Soc Work.

[7] Alfieri S, Sironi E, Marta E, Rosina A, Marzana D (2015). Young Italian NEETs (not in employment, education, or training) and the influence of their family background. Eur J Psychol.

[8] Backman O, Nilsson A (2016). Long-term consequences of being not in employment, education or training as a young adult. Stability and change in three Swedish birth cohorts. Eur Soc.

[9] Bynner J (2012). Policy reflections guided by longitudinal study, youth training, social exclusion, and more recently NEET. Br J Educ Stud.

[10] Ralston K, Feng Z, Everington D, Dibben C (2016). Do young people not in education, employment or training experience long-term occupational scarring? A longitudinal analysis over 20 years of follow-up. Contemp Soc Sci.

[11] Symonds J, Dietrich J, Chow A, Salmela-Aro K (2016). Mental health improves after transition from comprehensive school to vocational education or employment in england: a national cohort study. Dev Psychol.

[12] Benjet C, Hernandez-Montoya D, Borges G, Mendez E, Medina-Mora ME, Aguilar-Gaxiola S (2012). Youth who neither study nor work: mental health, education and employment. Salud Publica Mex.

[13] Nardi B, Lucarelli C, Talamonti M, Arimatea E, Fiori V, Moltedo-Perfetti A (2015). NEETs versus EETs: an observational study in Italy on the framework of the HEALTH25 European project. Res Post Compuls Educ.

[14] Goldman-Mellor S, Caspi A, Arseneault L, Ajala N, Ambler A, Danese A, Fisher H, Hucker A, Odgers C, Williams T, Wong C, Moffitt TE (2016). Committed to work but vulnerable: self-perceptions and mental health in NEET 18-year olds from a contemporary British cohort. J Child Psychol Psychiatry.

[15] Gutierrez-Garcia RA, Benjet C, Borges G, Rios EM, Medina-Mora ME (2017). NEET adolescents grown up: Eight-year longitudinal follow-up of education, employment and mental health from adolescence to early adulthood in Mexico City. Eur Child Adolesc Psychiatry.

[16] O’Dea B, Lee RSC, McGorry PD, Hickie IB, Scott J, Hermens DF, Mykeltun A, Purcell R, Killackey E, Pantelis C, Amminger GP, Glozier N (2016). A prospective cohort study of depression course, functional disability, and NEET status in help-seeking young adults. Soc Psychiatry Psychiatr Epidemiol.

[17] Baggio S, Iglesias K, Deline S, Studer J, Henchoz Y, Mohler-Kuo M, Gmel G (2015). Not in education, employment, or training status among young Swiss men. Longitudinal associations with mental health and substance use. J Adolesc Health.

[18] Power E, Clarke M, Kelleher I, Coughlan H, Lynch F, Connor D, Fitzpatrick C, Harley M, Cannon M (2015). The association between economic inactivity and mental health among young people: a longitudinal study of young adults who are not in employment, education or training. Ir J Psychol Med.

[19] Henderson JL, Hawke LD, Chaim G (2017). Not in employment, education or training: mental health, substance use, and disengagement in a multi-sectoral sample of service-seeking Canadian youth. Child Youth Serv Rev.

[20] Bynner J, Parsons S (2002). Social exclusion and the transition from school to work: the case of young people not in education, employment, or training (NEET). J Vocat Behav.

[21] Reneflot A, Evensen M (2014). Unemployment and psychological distress among young adults in the Nordic countries: a review of the literature. Int J Soc Welf.

[22] Vancea M, Utzet M (2017). How unemployment and precarious employment affect the health of young people: a scoping study on social determinants. Scand J Public Health.

[23] Bowman S, McKinstry C, McGorry P (2017). Youth mental ill health and secondary school completion in Australia: time to act. Early Interv Psychiatry.

[24] Esch P, Bocquet V, Pull C, Couffignal S, Lehnert T, Graas M, Fond-Harmant L, Ansseau M (2014). The downward spiral of mental disorders and educational attainment: a systematic review on early school leaving. BMC Psychiatry.

[25] Stroup DF, Berlin JA, Morton SC, Olkin I, Williamson GD, Rennie D, Moher D, Becker BJ, Sipe TA, Thacker SB (2000). Meta-analysis of observational studies in epidemiology: a proposal for reporting. Meta-analysis Of Observational Studies in Epidemiology (MOOSE) group. JAMA.

[26] Gariepy, G., Iyer, S.N. The mental health of youth not employed or in education: a systematic review of current knowledge. PROSPERO: International prospective register of systematic reviews. 2018. CRD42018087446. Available from: https://www.crd.york.ac.uk/prospero/display_record.php?ID=CRD42018087446

[27] Batini F, Corallino V, Toti G, Bartolucci M (2017). NEET: a phenomenom yet to be explored. Interchange.

[28] Veritas Health Innovation Covidence systematic review software, Melbourne, Australia. http://www.covidence.org. Accessed 01 May 2020

[29] Wells GA, Shea B, O’Connell D, Peterson J, Welch V, Losos M, Tugwell P The Newcastle–Ottawa Scale (NOS) for assessing the quality of nonrandomised studies in meta-analyses. Ottawa Health Research Institute. http://www.ohri.ca/programs/clinical_epidemiology/oxford.htm. Accessed 01 Jan 2021

[30] Tanner-Smith EE, Tipton E, Polanin JR (2016). Handling complex meta-analytic data structures using robust variance estimates: a tutorial in R. J Dev Life Course Criminol.

[31] Tipton E (2015). Small sample adjustments for robust variance estimation with meta-regression. Psychol Methods.

[32] Lin E, Tong T, Chen Y, Wang Y (2020) Fixed-effects model: the most convincing model for meta-analysis with few studies. arXiv preprint arXiv: 2002. 04211

[33] Peters JL, Sutton AJ, Jones DR, Abrams KR, Rushton L, Moreno SG (2010). Assessing publication bias in meta-analyses in the presence of between-study heterogeneity. J R Stat Soc A Stat Soc.

[34] Balduzzi S, Rücker G, Schwarzer G (2019). How to perform a meta-analysis with R: a practical tutorial. Evid Based Ment Health.

[35] Viechtbauer W (2010). Conducting meta-analyses in R with the meta for package. J Stat Softw.

[36] Nardi B, Arimatea E, Giunto P, Lucarelli C, Nocella S, Bellantuono C (2013). not employed in education or training (NEET) adolescents with unlawful behaviour: an observational study. J Psychopathol.

[37] Cairns AJ, Kavanagh DJ, Dark F, McPhail SM (2018). Comparing predictors of part-time and no vocational engagement in youth primary mental health services: a brief report. Early Interv Psychiatry.

[38] O'Dea B, Glozier N, Purcell R, McGorry PD, Scott J, Feilds KL, Hermens DF, Buchanan J, Scott EM, Yung AR, Killacky E, Guastella AJ, Hickie IB (2014). A cross-sectional exploration of the clinical characteristics of disengaged (NEET) young people in primary mental healthcare. BMJ Open.

[39] Gutierrez-Garcia RA, Benjet C, Borges G, Mendez Rios E, Medina-Mora ME (2018). Emerging adults not in education, employment or training (NEET): socio-demographic characteristics, mental health and reasons for being NEET. BMC Public Health.

[40] Gariépy G, Iyer S (2019). The mental health of young canadians who are not working or in school. Can J Psychiat.

[41] López-López JA, Kwong AS, Washbrook E, Pearson RM, Tilling K, Fazel MS, Kidger J, Hammerton G (2019). Trajectories of depressive symptoms and adult educational and employment outcomes. BJPsych Open.

[42] Bania EV, Eckhoff C, Kvernmo S (2019). Not engaged in education, employment or training (NEET) in an Arctic sociocultural context: the NAAHS cohort study. BMJ Open.

[43] Hammerton G, Murray J, Maughan B, Barros FC, Gonçalves H, Menezes AMB, Wehrmeister FC, Hickman M, Heron J (2019). Childhood behavioural problems and adverse outcomes in early adulthood: a comparison of Brazilian and British birth cohorts. J Dev Life Course Criminol.

[44] Rodwell L, Romaniuk H, Nilsen W, Carlin JB, Lee KJ, Patton GC (2018). Adolescent mental health and behavioural predictors of being NEET: a prospective study of young adults not in employment, education, or training. Psychol Med.

[45] Stea TH, de Ridder K, Haugland SH (2019). Comparison of risk-behaviors among young people who are not in education, employment or training (NEET) versus high school students. A cross-sectional study. Norsk Epidemiologi.

[46] Basta M, Karakonstantis S, Koutra K, Dafermos V, Papargiris A, Drakaki M, Tzagkarakis S, Vgontzas A, Simos P, Papadakis N (2019). NEET status among young Greeks: association with mental health and substance use. J Affect Disord.

[47] Hale DR, Bevilacqua L, Viner RM (2015). Adolescent health and adult education and employment: a systematic review. Pediatrics.

[48] Manhica H, Lundin A, Danielsson A-K (2019). Not in education, employment, or training (NEET) and risk of alcohol use disorder: a nationwide register-linkage study with 485 839 Swedish youths. BMJ Open.

[49] Hale DR, Viner RM (2018). How adolescent health influences education and employment: investigating longitudinal associations and mechanisms. J Epidemiol Community Health.

[50] Stea TH, Abildsnes E, Strandheim A, Haugland SH (2019). Do young people who are not in education, employment or training (NEET) have more health problems than their peers? A cross-sectional study among Norwegian adolescents. Norsk Epidemiologi.

[51] Hammarström A, Ahlgren C (2019). Living in the shadow of unemployment—an unhealthy life situation: a qualitative study of young people from leaving school until early adult life. BMC Public Health.

[52] Mitchell DP, Betts A, Epling M (2002). Youth employment, mental health and substance misuse: a challenge to mental health services. J Psychiatr Ment Health Nurs.

[53] Brunet S (2018). The transition from school to work: the NEET (not in employment, education or training) indicator for 25-to 29-year-old women and men in Canada. Education Indicators in Canada: Fact Sheet.

[54] Eurofound (2016). Exploring the diversity of NEETs.

[55] Holte BH (2018). Counting and meeting NEET young people: methodology, perspective and meaning in research on marginalized youth. Young.

[56] Bloom DE, Cafiero E, Jané-Llopis E, Abrahams-Gessel S, Bloom LR, Fathima S, Feigl AB, Gaziano T, Hamandi A, Mowafi M, O’Farrell D, Ozaltin E, Pandya A, Prettner K, Rosenberg L, Seligman B, Stein AZ, Weinstein C, Weiss J (2012). The global economic burden of noncommunicable diseases.

[57] Rodwell L, Romaniuk H, Nilsen W, Carlin JB, Lee K, Patton GC (2018). Adolescent mental health and behavioural predictors of being NEET: a prospective study of young adults not in employment, education, or training. Psychol Med.

[58] Fusar-Poli P (2019). Integrated mental health services for the developmental period (0 to 25 years): a critical review of the evidence. Front Psychiatry.

[59] Malla A, Iyer S, McGorry P, Cannon M, Coughlan H, Singh S, Jones P, Joober R (2016). From early intervention in psychosis to youth mental health reform: a review of the evolution and transformation of mental health services for young people. Soc Psychiatry Psychiatr Epidemiol.

