# Supplementary material for: The mental health of young people who are not in education, employment, or training: a systematic review and meta-analysis
Source: Soc Psychiatry Psychiatr Epidemiol. 2021 Dec 21;57(6):1107–21. doi: 10.1007/s00127-021-02212-8 (PMC8687877; doi:10.1007/s00127-021-02212-8)
Supplement: Supplementary file 1 — Supplementary file1 (PDF 775 KB) [file 127_2021_2212_MOESM1_ESM.pdf]

The mental health of young people who are not in employment, education, or training: A systematic review and meta-analysis

Geneviève Gariépy, Ph.D., Sofia Danna, M.Sc., Lisa Hawke, Ph.D., Joanna Henderson, Ph.D., Srividya N. Iyer, Ph.D.

Supplementary material

Appendix 1. Example of Medline database search strategy

Appendix 2. Data extraction form

Appendix 3. Quality assessment description and scale

Appendix 4. Detailed summary of findings by study

Appendix 5. Quality assessment of the selected studies

Appendix 6. Forest plot: NEET status and the aggregate group of mental-ill health measures

Appendix 7. Forest plot: NEET status and the aggregate group of substance use problems

Appendix 8. Subgroup analyses with aggregate groups

Appendix 9. Summary table of significant findings of studies by directionality of association and type of mental or substance use disorder or symptoms

Appendix 1. Example of Medline database search strategy

exp mental disorders/ OR exp Mental Health/ OR ("psychiatric disorder* " OR "mental disorder*" OR "mental illness*" OR "mental health" OR depression OR depressive OR anxiety OR anxious OR mood OR bipolar OR "substance abuse" OR "substance use" OR "substance misuse" OR "substance dependence" OR "alcohol abuse" OR "alcohol use" OR "alcohol misuse" OR "alcohol dependence" OR addictive OR addiction OR psychotic OR psychosis OR schizo* OR "personality disorder*" OR "conduct disorder*" OR phobia OR phobic OR OCD OR "obsessive compulsive" OR PTSD OR "post-traumatic stress*" OR "eating disorder*" OR anorex* OR bulimi* OR "self-harm" OR "self-injury" OR "self-mutilat*" OR suicid* OR "psychological distress" OR "psychological well-being" OR "mental well-being" OR "subjective well-being" OR "emotional well-being" OR bully* OR "behavioral problem*" OR "behavioral problem*" OR "externalizing problem*" OR "externalizing disorder*" OR "internalizing problem*" OR "internalizing disorder*" OR "psychological health" OR "psychological symptom*").ab,ti.

AND

"NEET".ab,ti OR (school* adj2 work).ab,ti. OR (school* adj2 employ*).ab,ti. OR (school* adj2 unemploy*).ab,ti. OR (educa* adj2 work).ab,ti. OR (educa* adj2 employ*).ab,ti. OR (educa* adj2 unemploy*).ab,ti. OR (training adj2 work) .ab,ti. OR (training adj2 employ*).ab,ti. OR (training adj2 unemploy*).ab,ti. OR ((employment/ OR unemployment/) AND (Educational Status/ OR Student Dropouts/))

AND

exp Young Adult/ OR exp Adolescent/ OR (young OR youth* OR adolescen* OR teen*).ab,ti.

Appendix 2. Data extraction form

The data extraction form included the following items.

ITEMS	DETAILS
**Title**	Full title of article or report
**First author**	Name of first author
**Year**	Publication year
**Country**	Country where study took place
**Study design**	Cross-sectional; cohort; case-control; other, specify
**Target population**	Population; clinical help-seeking sample from a catchment-area; clinical help-seeking sample from a service
**Sample size**	Sample size used in analysis
**Sample characteristics**	Age range; specific subpopulation; other details
**Date of data collection**	Date; number of time points (if longitudinal study)
**NEET measure**	Definition and measurement (age range, variables used, etc.); timeframe (e.g., current, past six months); other details (e.g., excluded military workers)
**Mental health measure**	Type; instrument or scale; timeframe (e.g., past year, lifetime); type of measure (continuous or binary)
**Analysis**	Type of statistical analysis (e.g., logistic regression, latent class)
**Covariates**	List of covariates included in the final analysis
**Temporality**	Association between NEET status before, concurrently, or after measure of mental health
**Estimates of association**	Measure of association (OR, RR, etc.) and 95% confidence intervals or p-value
**Comments**	Any additional comments

Appendix 3. Quality assessment description and scale

We used a modified version of the Newcastle–Ottawa Scale (Wells et al.) that we previously developed (Gariépy, Nitka, & Schmitz, 2010; Smith et al., 2013) to critically appraise potential biases and the overall quality of studies. The modified scale includes eight items about comparability, selection bias, information bias, and control of at least three important confounders. We evaluated if the components of the checklist were met (yes; no; not reported) and appraised the overall quality of evidence of the study (poor; moderate; high). High-quality studies had satisfactory ratings on all components and up to one component that was not reported. Medium quality studies had unsatisfactory ratings on up to one component, or up to two components that were not reported. Low quality studies had unsatisfactory rating on more than one component, or more than two components that were not reported.

ITEMS	DETAILS
**SELECTION**	
1. Representativeness	Were study participants representative of the study base?
2. Participation rate	Was the overall participation rate > 60%?
3. Consistency across groups	Were people with different levels of NEET/mental health drawn from the same population?
4. Minimal risk of selection due to lost to follow-up	Was lost to follow-up unlikely to introduce bias? (Cohort study)
**MEASUREMENT**	
5. NEET measure	Was NEET status assessed using a clear or valid approach?
6. Mental health measure	Was mental health assessed using a validated instrument?
7. Same measurements across participants	Were NEET status and mental health assessed in the same way for the entire study sample?
**CONFOUNDING**	
8. Confounders	Did the study controlled for at least three important confounders?

References

Gariépy, G., Nitka, D., & Schmitz, N. (2010). The association between obesity and anxiety disorders in the population: A systematic review and meta-analysis. *International Journal of Obesity*, *34*(3), 407-19.

Smith, K. J., Beland, M., Clyde, M., Gariépy, G., Page, V., Badawi, G., … Schmitz, N. (2013). Association of diabetes with anxiety: A systematic review and meta-analysis. *Journal of Psychosomatic Research*, *74*(2), 89-99.

Wells, G., Shea, B., O'Connell, D., Peterson, J., Welch, V., Losos, M., & Tugwell, P. The Newcastle-Ottawa Scale (NOS) for assessing the quality of nonrandomised studies in meta-analyses. Retrieved from: http://www.ohri.ca/programs/clinical_epidemiology/oxford.htm.

Appendix 4. Detailed summary of findings by study

Study	Mental health measures	Directionality of association	Results
**Baggio et al 2015**	General mental health	MH<-->NEET	Beta coefficient NA, P>0.05
	Depressive symptoms	MH<-->NEET	Beta coefficient NA, P<0.001
	Binge drinking	MH<-->NEET	Beta coefficient NA, P>0.05
	Cannabis use disorder	MH<-->NEET	Beta coefficient NA, P<0.001
	Depressive symptoms	NEET-->MH	Beta coefficient 0.093, P>0.05
	Binge drinking	NEET-->MH	Beta coefficient 0.014, P>0.05
	Cannabis use disorder	NEET-->MH	Beta coefficient NA, P>0.05
	General mental health	NEET-->MH	Beta coefficient 0.028, P>0.05
	Depressive symptoms	MH-->NEET	OR 1.11, P<0.001
	Binge drinking	MH-->NEET	OR NA, P>0.05
	Cannabis use disorder	MH-->NEET	OR 1.09, P<0.001
	General mental health	MH-->NEET	OR 1.11, P<0.001
**Bania et al 2019**	Emotional problems	MH-->NEET	Men: OR 0.88, 95% CI 0.81, 0.97
	Emotional problems	MH-->NEET	Women: OR 1.04, 95% CI 0.97, 1.11
	Conduct problems	MH-->NEET	Men: OR 1.17, 95% CI 1.07, 1.28
	Conduct problems	MH-->NEET	Women: OR 1.25, 95% CI 1.17, 1.33
**Basta et al 2019**	Psychological distress	MH<-->NEET	All: OR 0.98, 95% CI 0.96, 1.01
	Psychological distress	MH<-->NEET	Men: OR 0.99, 95% CI 0.96, 1.03
	Psychological distress	MH<-->NEET	Women: OR 0.98, 95% CI 0.95, 1.02
	Anxiety symptoms	MH<-->NEET	All: OR 1.03, 95% CI 0.99, 1.06
	Anxiety symptoms	MH<-->NEET	Men: OR 1.01, 95% CI 0.96, 1.03
	Anxiety symptoms	MH<-->NEET	Women: OR 1.05, 95% CI 1.01, 1.10
	Drug use	MH<-->NEET	All: OR 0.80, 95% CI 0.58, 1.1
	Drug use	MH<-->NEET	Men: OR 0.99, 95% CI 0.67, 1.48
	Drug use	MH<-->NEET	Women: OR 0.52, 95% CI 0.28, 0.94
**Benjet et al 2012**	Mood disorder	MH<-->NEET	OR 2.7, 95% CI 1.8, 4.2
	Anxiety disorder	MH<-->NEET	OR 1.3, 95% CI 0.9, 1.8
	Substance use disorder	MH<-->NEET	OR 4.4, 95% CI 2.6, 7.6
	Behavioral disorder	MH<-->NEET	OR 2.1, 95% CI 1.5, 2.8
	Any disorder	MH<-->NEET	OR 1.7, 95% CI 1.3, 2.3
	Suicidal ideation	MH<-->NEET	OR 1.8, 95% CI 1.1, 2.8
	Suicidal plan	MH<-->NEET	OR 2.4, 95% CI 1.4, 4.1
	Suicidal attempt	MH<-->NEET	OR 3.6, 95% CI 2.0, 6.4
**Bynner & Parsons 2002**	Psychological distress	NEET-->MH	Men: OR 2.20, P>0.05
	Psychological distress	NEET-->MH	Women: OR 1.69, P>0.05
**Cairns et al 2018**	Psychological distress	MH<-->NEET	Beta coefficient 0.03, 96%CI -0.02, 0.07
	History of psychiatric diagnosis	MH<-->NEET	Beta coefficient 0.61, 96%CI -0.21, 1.43
	History of drug use	MH<-->NEET	Beta coefficient 1.11, 96%CI 0.32, 1.91
**Gariépy & Iyer 2019**	Depressive disorder	MH<-->NEET	OR 1.7, 95% CI 1.1, 2.6
	Bipolar disorder	MH<-->NEET	OR 2.3, 95% CI 1.0, 5.5
	Generalized anxiety disorder	MH<-->NEET	OR 2.6, 95% CI 1.4, 5.1
	Alcohol use disorder	MH<-->NEET	OR 1.0, 95% CI 0.6, 1.7
	Cannabis disorder	MH<-->NEET	OR 1.0, 95% CI 0.5, 2.0
	Drug use disorder	MH<-->NEET	OR 3.2, 95% CI 1.3, 7.8
	Suicidal ideation	MH<-->NEET	OR 1.8, 95% CI 1.0, 3.1
**Goldman-Mellor et al 2016**	Depressive disorder	MH<-->NEET	OR 1.68, 95% CI 1.33, 2.13
	Generalized anxiety disorder	MH<-->NEET	OR 1.84, 95% CI 1.19, 2.86
	Alcohol dependence	MH<-->NEET	OR 1.42, 95% CI 1.01, 2.01

	Cannabis dependence	MH<-->NEET	OR 3.93, 95% CI 2.31, 6.68
	Conduct disorder	MH<-->NEET	OR 1.46, 95% CI 1.12, 1.89
	Depressive disorder	MH-->NEET	OR 2.57, 95% CI 1.40, 4.72
	Anxiety disorder	MH-->NEET	OR 1.38, 95% CI 0.81, 2.37
	Harmful substance use	MH-->NEET	OR 1.89, 95% CI 1.29, 2.77
	Suicidal behaviors	MH-->NEET	OR 3.30, 95% CI 2.07, 5.27
**Gutierrez-Garcia et al 2017**			*NEET vs those in school only*
	Mood disorder	NEET-->MH	OR 1.67, 95% CI 1.12, 1.90
	Anxiety disorder	NEET-->MH	OR 0.40, 95% CI 0.10, 1.65
	Behavioral disorder	NEET-->MH	OR 0.83, 95% CI 0.45, 1.50
	Alcohol use disorder	NEET-->MH	OR 1.19, 95% CI 1.14, 1.38
	Drug use disorder	NEET-->MH	OR 1.03, 95% CI 0.71, 1.50
	Suicidal ideation	NEET-->MH	OR 1.94, 95% CI 1.16, 3.31
	Suicidal plan	NEET-->MH	OR 1.18, 95% CI 1.07, 2.38
	Suicidal attempt	NEET-->MH	OR 2.40, 95% CI 1.30, 4.31
**Gutierrez-Garcia et al 2018**			*NEET vs those in school only*
	Mood disorder	MH<-->NEET	OR 0.73, 95% CI 0.39, 1.34
	Anxiety disorder	MH<-->NEET	OR 0.75, 95% CI 0.35, 1.62
	Behavioral disorder	MH<-->NEET	OR 0.79, 95% CI 0.37, 1.67
	Alcohol use disorder	MH<-->NEET	OR 1.38, 95% CI 1.02, 1.45
	Substance use disorder	MH<-->NEET	OR 1.73, 95% CI 1.02, 3.41
	Drug use disorder	MH<-->NEET	OR 0.90, 95% CI 0.38, 2.16
	Suicidal ideation	MH<-->NEET	OR 1.17, 95% CI 0.52, 2.65
	Suicidal plan	MH<-->NEET	OR 0.12, 95% CI 0.01, 1.20
	Suicidal attempt	MH<-->NEET	OR 2.75, 95% CI 1.79, 4.25
**Hale & Viner 2018**	Psychological distress	MH-->NEET	Men: OR 1.72, 95% CI 1.24, 2.41
	Psychological distress	MH-->NEET	Women: OR 1.49, 95% CI 1.11, 1.99
**Hammerton et al 2019**	Oppositional problem, Brazil	MH-->NEET	OR 1.24, 95% CI 1.04, 1.47
	Conduct problem, Brazil	MH-->NEET	OR 1.38, 95% CI 1.13, 1.70
	Oppositional problem, UK	MH-->NEET	OR 1.22, 95% CI 0.78,1.91
	Conduct problem, UK	MH-->NEET	OR 3.04, 95% CI 1.99, 4.65
**Henderson et al 2017**	Internalizing disorders	MH<-->NEET	Total: OR 1.15, 95% CI 0.95, 1.38
	Internalizing disorders	MH<-->NEET	Age<18: OR 0.95, 95% CI 0.72, 1.26
	Internalizing disorders	MH<-->NEET	Age≥18: OR 1.33, 95% CI 1.00, 1.75
	Internalizing disorders	MH<-->NEET	Men: OR 1.39, 95% CI 1.08, 1.78
	Internalizing disorders	MH<-->NEET	Women: OR 1.08, 95% CI 0.80, 1.45
	Externalizing disorders	MH<-->NEET	Total: OR 0.90, 95% CI 0.76, 1.08
	Externalizing disorders	MH<-->NEET	Age<18: OR 1.18, 95% CI 0.90, 1.56
	Externalizing disorders	MH<-->NEET	Age≥18: OR 1.13, 95% CI 0.87, 1.46
	Externalizing disorders	MH<-->NEET	Men: OR 0.93, 95% CI 0.73, 1.19
	Externalizing disorders	MH<-->NEET	Women: OR 0.87, 95% CI 0.67, 1.12
	Substance use disorder	MH<-->NEET	Total: OR 1.94, 95% CI 1.63, 2.31
	Substance use disorder	MH<-->NEET	Age<18: OR 1.44, 95% CI 1.21, 1.88
	Substance use disorder	MH<-->NEET	Age≥18: OR 2.19, 95% CI 1.68, 2.84
	Substance use disorder	MH<-->NEET	Men: OR 1.83, 95% CI 1.43, 2.34
	Substance use disorder	MH<-->NEET	Women: OR 2.05, 95% CI 1.58, 2.66
**López-López et al 2019**	Depressive symptoms	MH-->NEET	*Persistent adolescent depression vs low/no*<br>OR 5.17, 95% CI 1.95, 13.70
**Manhica et al 2019**	Alcohol use disorder	NEET-->MH	HR 1.30, 95% CI 1.11, 1.51
**Nardi et al 2015**	Symptoms of nervousness, mood swings or thoughts of suicide	MH<-->NEET	No significant differences between NEET and non-NEET group

**Nardi et al 2013**	Psychiatric disorders diagnosed by psychiatric services	MH<-->NEET	Significant difference (p < 0.01) between NEET and non-NEET group
**O'Dea et al 2016**	Depressive disorders	MH-->NEET	OR 1.11, 95 % CI 1.02, 1.20
	Generalized anxiety disorders	MH-->NEET	OR NA, P>0.05
	Depressive disorders	NEET-->MH	OR 1.94, 95 % CI 0.17, 21.6
	Generalized anxiety disorders	NEET-->MH	OR NA, P>0.05
**O'Dea et al 2014**	Mood disorder	MH<-->NEET	OR 1.10, 95% CI 1.05,1.15
	Anxiety disorder	MH<-->NEET	OR NA, P>0.05
	Alcohol use disorder	MH<-->NEET	OR NA, P>0.05
	Cannabis use disorder	MH<-->NEET	OR 1.22, 95% CI 0.72, 2.05
**Power et al 2015**	Depressive disorder (lifetime)	MH<-->NEET	OR NA, P>0.05
	Anxiety disorder (lifetime)	MH<-->NEET	OR 2.4, 95% CI 1.0, 5.9
	Alcohol use disorder (lifetime)	MH<-->NEET	OR NA, P>0.05
	Drug use disorder (lifetime)	MH<-->NEET	OR NA, P>0.05
	Suicidal ideation (lifetime)	MH<-->NEET	OR NA, P>0.05
	Suicidal attempts (lifetime)	MH<-->NEET	OR 3.1, 95% CI 1.0, 9.3
	Any lifetime disorder	MH<-->NEET	OR 2.9, 95% CI 1.1, 7.2
	Any current disorder	MH<-->NEET	OR NA, P>0.05
	Any diagnosis in childhood	MH-->NEET	OR 4.0, 95% CI 1.7, 9.4
	Any diagnosis in adolescence	MH-->NEET	OR 3.1, 95% CI 1.4, 7.0
**Rodwell et al 2018**	Common mental disorders	MH-->NEET	OR 1.60, 95% CI 1.07, 2.40
	Disruptive behavior disorder	MH-->NEET	OR 1.71, 95% CI 1.15, 2.55
	Cannabis use disorder	MH-->NEET	OR 1.74, 95% CI 1.10, 2.75
	Heavy binge drinking	MH-->NEET	OR 0.80, 95% CI 0.48, 1.34
**Stea, Abildsnes et al 2019**	Psychological distress	MH<-->NEET	Men: OR NA, P>0.05
		MH<-->NEET	Women: OR 2.40, 95% CI 1.00, 5.20
**Stea, de Ridder et al 2019**	Cannabis use	MH<-->NEET	OR 2.20; 95% CI 1.10, 4.30
**Symonds et al 2016**			*NEET vs those in school:*
	Depressive symptoms	MH-->NEET	Beta coefficient 0.10, P<0.05
	Anxiety symptoms	MH-->NEET	Beta coefficient 0.07, P>0.05
	Positive mental functioning	MH-->NEET	Beta coefficient -0.06, P<0.05
			NEET vs those at work:
	Depressive symptoms	MH-->NEET	Beta coefficient 0.07, P>0.05
	Anxiety symptoms	MH-->NEET	Beta coefficient 0.08, P<0.05
	Positive mental functioning	MH-->NEET	Beta coefficient -0.05, P<0.05

NEET: Not in education, employment, or training; MH: Mental health; NA data was not available

Appendix 5. Quality assessment of the selected studies

Study	Participants representative of study base	Participation rate > 60%	Participants drawn from the same population	Clear NEET measure	Valid mental health measure	Outcome assessed the same across sample	Minimal risk of selection due to lost to follow-up	Controlled for ≥ three confounders	Overall quality
**Baggio et al 2015**	Yes	Yes	Yes	Yes	Yes	Yes	Yes	Yes	High
**Bania et al 2019**	Yes	Yes	Yes	Yes	Yes	Yes	Yes	Yes	High
**Basta et al 2019**	Not reported	Yes	Yes	Yes	Yes	Yes	Not applicable	Yes	High
**Benjet et al 2012**	Yes	Yes	Yes	Yes	Yes	Yes	Not applicable	Yes	High
**Bynner & Parsons 2002**	Not reported	Not reported	Yes	Yes	Yes	Yes	Yes	Yes	Moderate
**Cairns et al 2018**	Not reported	Yes	Yes	Yes	Yes	Yes	Not applicable	Yes	High
**Gariépy & Iyer 2019**	Yes	Yes	Yes	Yes	Yes	Yes	Not applicable	Yes	High
**Goldman-Mellor et al 2016**	Yes	Yes	Yes	Yes	Yes	Yes	Yes	Yes	High
**Gutierrez-Garcia et al 2017**	No	Yes	Yes	Yes	Yes	Yes	No	Yes	Low
**Gutierrez-Garcia et al 2018**	No	Yes	Yes	Yes	Yes	Yes	Not applicable	Yes	Moderate
**Hale & Viner 2018**	Yes	Yes	Yes	Yes	Yes	Yes	No	No	Moderate
**Hammerton et al 2019**	Yes	Yes	Yes	Yes	Yes	Yes	No	Yes	Moderate
**Henderson et al 2017**	Not reported	Yes	Yes	Yes	Yes	Yes	Not applicable	No	Moderate
**López-López et al 2019**	Yes	Yes	Yes	Yes	Yes	Yes	No	Yes	Moderate
**Manhica et al 2019**	Yes	Yes	Yes	Yes	Yes	Yes	Yes	Yes	High

**Nardi et al 2015**	No	Not reported	No	No	No	Not reported	Not applicable	No	Low
**Nardi et al 2013**	Yes	Yes	Yes	No	Yes	Yes	Not applicable	No	Low
**O'Dea et al 2016**	Yes	Not reported	Yes	Yes	Yes	Yes	No	Yes	Moderate
**O'Dea et al 2014**	Yes	Not reported	Yes	Yes	Yes	Yes	Not applicable	Yes	High
**Power et al 2015**	Yes	No	Yes	No	Yes	No	Yes	No	Low
**Rodwell et al 2018**	Yes	Not reported	Yes	Yes	Yes	Yes	Yes	Yes	High
**Stea, Abildsnes et al 2019**	Yes	Not reported	No	Yes	Yes	Yes	Not applicable	No	Low
**Stea, de Ridder et al 2019**	Yes	Not reported	No	Yes	Yes	Yes	Not applicable	Yes	Moderate
**Symonds et al 2016**	Yes	Not reported	Yes	Yes	Yes	Yes	No	Yes	Moderate

Appendix 6. Forest plot: NEET status and the aggregate group of mental-ill health measures

Forest Plot

Studies	Log OR	Variance
**Baggio et al 2015**		
General mental health	0.104	0.032
Depressive symptoms	0.104	0.032
**Bania et al 2019**		
Emotional problems	–0.029	0.028
Conduct problems	0.200	0.027
**Basta et al 2019**		
Psychological distress	–0.020	0.013
Anxiety symptoms	0.030	0.017
**Benjet et al 2012**		
Mood disorder	0.993	0.216
Anxiety disorder	0.262	0.177
Behavioral disorder	0.742	0.159
**Bynner & Parsons 2002**		
Psychological distress	0.578	0.332
**Gariépy & Iyer 2019**		
Depressive disorder	0.531	0.219
Generalized anxiety disorder	0.956	0.330
**Goldman–Mellor et al 2016**		
Depressive disorder	0.519	0.120
Generalized anxiety disorder	0.610	0.224
Conduct disorder	0.378	0.133
**Hale & Viner 2018**		
Psychological distress	0.453	0.116
**Hammerton et al 2019**		
Conduct problem	0.322	0.104
Conduct problem	1.112	0.217
**Henderson et al 2017**		
Internalizing disorders	0.140	0.095
Externalizing disorders	–0.105	0.090
**López–López et al 2019**		
Depressive symptoms	1.643	0.497
**O'Dea et al 2014**		
Mood disorder	0.095	0.023
**Power et al 2015**		
Anxiety disorder (lifetime)	0.875	0.453
**Rodwell et al 2018**		
Disruptive behaviour disorder	0.536	0.203
**Stea, Abildsnes et al 2019**		
Psychological distress	0.875	0.421

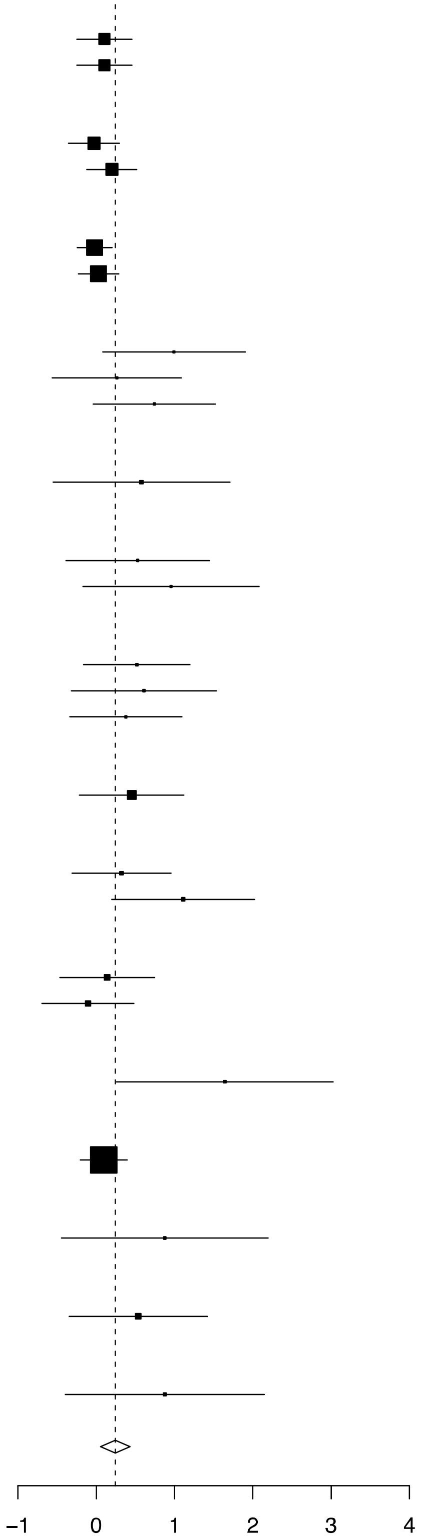

The size of the box around the estimates represents the relative sample size of the study. The bars represent the 95% confidence intervals of the estimate

Hammerton et al. 2019 includes two Conduct problem samples, one from the UK and the other from Brazil.

Appendix 7. Forest plot: NEET status and the aggregate group of substance use problems

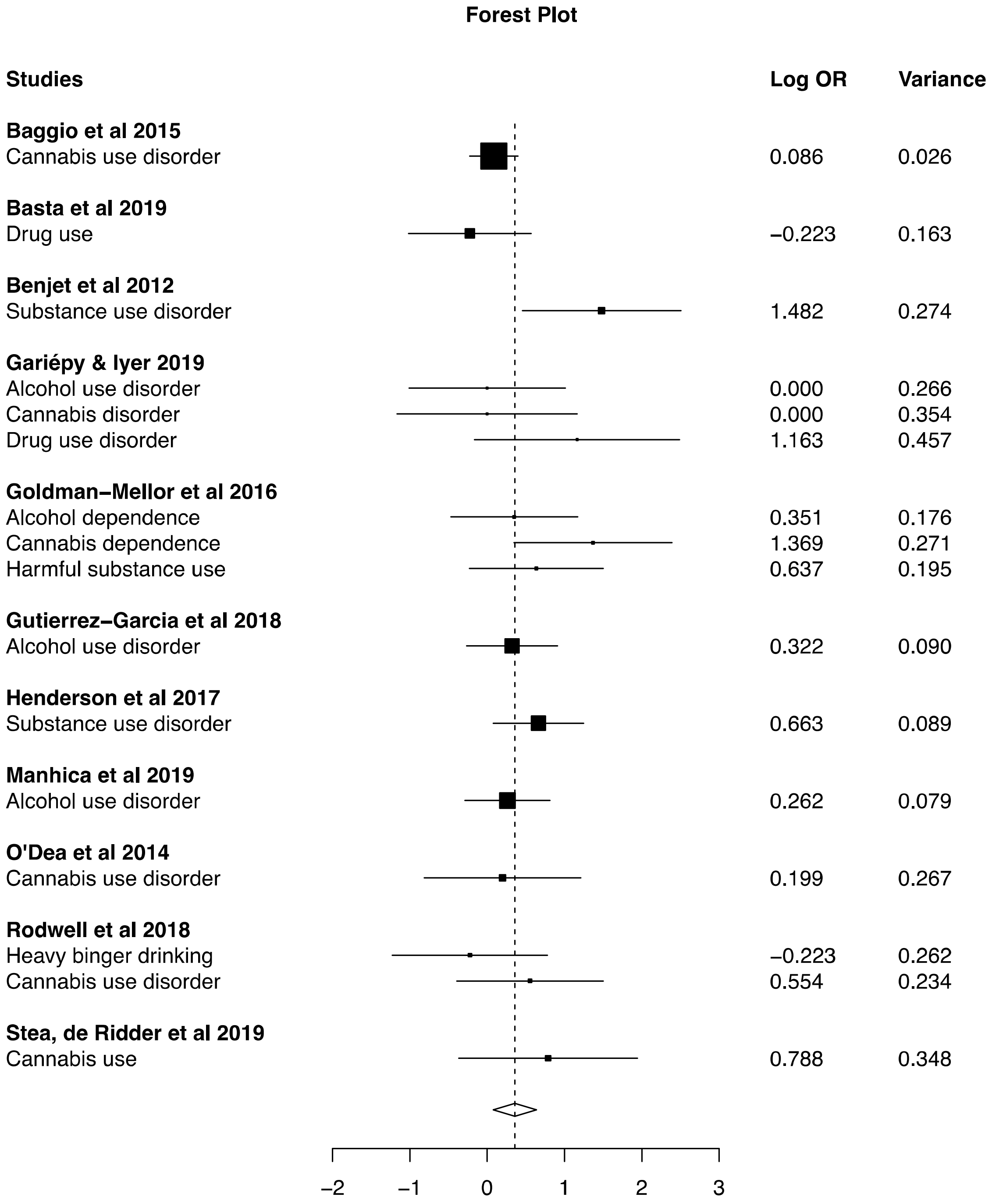

The size of the box around each estimates represents the relative sample size of the study. The bars represent the 95% confidence intervals of the estimate.

Appendix 8. Subgroup analyses with aggregate groups

Analysis	*k*	*n*	Estimate; 95% CI	*df*
**Mental health**				
Directionality: MH -> NEET	8	12	1.33; CI 1.01, 1.74	4.66
Directionality: NEET -> MH	3	5	1.32; CI 0.20, 8.50	1.53
Age: <18	4	8	1.20; CI 0.60, 2.42	1.64
Age: ≥18	7	13	1.28; CI 0.88, 1.86	2.85
Gender: girl	6	9	1.12; CI 0.80, 1.55	1.98
Gender: boy	5	8	1.07; CI 0.75, 1.53	1.75
**Substance use**				
Directionality: MH -> NEET	3	4	1.16; CI 0.39, 3.49	1.23
Directionality: NEET -> MH	2	3	1.22; CI 0.45, 3.32	1
Age: <18	2	2	2.34; CI 0.00, 2670.44	1
Age: ≥18	5	9	1.40; CI 0.78, 2.51	3
Gender: girl	2	2	1.10; CI 0.00, 6438.17	1
Gender: boy	2	2	1.43; CI 0.03, 65.37	1
**All measures**				
Directionality: MH -> NEET	9	19	1.39; CI 1.03, 1.86	5.41
Directionality: NEET -> MH	4	9	1.34; CI 0.92, 1.96	1.66
Age: <18	4	12	1.33; CI 0.65, 2.75	2.2
Age: ≥18	7	27	1.32; CI 0.83, 2.12	2.78
Gender: girl	6	11	1.19; CI 0.89, 1.61	2.7
Gender: boy	5	10	1.16; CI 0.80, 1.68	2.59

k = number of studies, *n* = number of estimates, *df* = degrees of freedom. Note: results are not reliable when *df*<4.

Appendix 9. Summary table of significant findings by directionality of association and type of mental or substance use disorder or symptoms

STUDY	Mood	Anxiety	Behavioral	Alcohol Use	Cannabis Use	Drug Use	Any Disorder	Suicidal Behaviors	Psychological Distress
**Baggio et al 2015**	**MH<-->NEET***<br>MH-->NEET<br>**NEET-->MH***			MH<-->NEET<br>MH-->NEET<br>NEET-->MH	MH<-->NEET<br>**MH-->NEET***<br>NEET-->MH				MH<-->NEET<br>**MH-->NEET***<br>NEET-->MH
**Bania et al 2019**			**MH-->NEET***						MH-->NEET
**Basta et al 2019**									MH<-->NEET
**Benjet et al 2012**	**MH<-->NEET ***	MH<-->NEET	**MH<-->NEET***			**MH<-->NEET ***	**MH<-->NEET***	**MH<-->NEET***	
**Bynner & Parsons 2002**									NEET-->MH
**Cairns et al 2018**						**MH<-->NEET***	MH-->NEET		MH<-->NEET
**Gariépy & Iyer 2019**	**MH<-->NEET***	**MH<-->NEET***		MH<-->NEET	MH<-->NEET	**MH<-->NEET***		**MH<-->NEET***	
**Goldman-Mellor et al 2016**	**MH<-->NEET***<br>**MH-->NEET***	**MH<-->NEET***<br>MH-->NEET	**MH<-->NEET***<br>**MH-->NEET***	**MH<-->NEET***	**MH<-->NEET***	**MH-->NEET***		**MH-->NEET***	
**Gutierrez-Garcia et al 2017**	**NEET-->MH***	NEET-->MH	NEET-->MH	**NEET-->MH***		**NEET-->MH***		**NEET-->MH***	
**Gutierrez-Garcia et al 2018**	MH<-->NEET	MH<-->NEET	MH<-->NEET	MH<-->NEET		MH<-->NEET		MH<-->NEET	
**Hale & Viner 2018**									**MH-->NEET***
**Hammerton et al 2019**			**MH-->NEET***						
**Henderson et al 2017**			MH<-->NEET			**MH<-->NEET***			MH<-->NEET
**López-López et al 2019**	**MH-->NEET***								
**Manhica et al 2019**				**NEET-->MH***					
**Nardi et al 2015**	MH<-->NEET	MH<-->NEET						MH<-->NEET	
**Nardi et al 2013**							**MH<-->NEET***<br>**MH-->NEET***		

**O'Dea et al 2016**	**MH-->NEET***	MH-->NEET<br>NEET-->MH							
**O'Dea et al 2014**	**MH<-->NEET***	MH<-->NEET		MH<-->NEET	MH<-->NEET				
**Power et al 2015**	MH<-->NEET	**MH<-->NEET***		MH<-->NEET		MH<-->NEET	MH<-->NEET<br>**MH<-->NEET***	**MH<-->NEET***	
**Rodwell et al 2018**			**MH-->NEET***	MH-->NEET	**MH-->NEET***		**MH-->NEET***		
**Stea, Abildsnes et al 2019**									**MH<-->NEET***
**Stea, de Ridder et al 2019**					**MH<-->NEET***				
**Symonds et al 2016**	**MH-->NEET***	**MH-->NEET***							**MH-->NEET***

Legend. Bold and * indicates statistically significant association (p>0.05). MH: mental health; NEET: not employed, in education, or training; MH<-->NEET: cross-sectional association between a mental health (MH) measure and being not employed, in education, or training (NEET); MH-->NEET: longitudinal association between a MH measure and a later NEET status; NEET-->MH: longitudinal association between a NEET status and a later MH measure.
